# Male mice are more susceptible than females to obesity‐induced muscle atrophy when fed a high‐fat, high‐cholesterol diet

**DOI:** 10.14814/phy2.71158

**Published:** 2026-10-30

**Authors:** Tingyi Mo, Shan Wang, Zhixin Li, Haobin Xu, Xiaokang Liufu, Ya Wang, Nageena Qayyum, Honghui Guo

**Affiliations:** ^1^ Department of Nutrition, School of Public Health Guangdong Medical University Zhanjiang China; ^2^ Dongguan Key Laboratory of Prevention and Treatment of Chronic Noncommunicable Diseases, School of Public Health Guangdong Medical University Dongguan China

**Keywords:** high‐fat and high‐cholesterol diet, mitochondrial function, muscle atrophy, obesity, oxidative phosphorylation, sexual dimorphism

## Abstract

Obesity‐related skeletal muscle atrophy poses a significant health burden, with marked sex differences in this condition. However, the mechanisms underlying these sex differences remain poorly understood. This study investigated whether and to what extent skeletal muscle quality was impaired in C57BL/6 mice fed a high‐fat, high‐cholesterol (HFHC) diet, focusing on sexual dimorphism. Male and female mice were assigned to control or HFHC groups for 40 weeks. The results showed that both sexes fed the HFHC diet developed obesity and dyslipidemia, with males exhibiting a more severe phenotype. Notably, males experienced more pronounced obesity‐induced muscle atrophy than females. This study further revealed that HFHC feeding disrupted physiological homeostasis in both sexes, leading to sexual dimorphism in gut microbial composition and short‐chain fatty acid levels, with males displaying a diminished capacity to maintain metabolic homeostasis. Furthermore, males exhibited more severe mitochondrial dysfunction and higher oxidative stress levels compared to females, manifesting as reduced muscle strength and upregulated atrophy‐related gene expression. Collectively, these findings indicate that long‐term HFHC feeding disrupts gut microbiota and glucolipid metabolic homeostasis, further impairing mitochondrial function and exacerbating muscle atrophy preferentially in males, whereas females are relatively protected through superior maintenance of physiological homeostasis, resulting in milder muscle atrophy.

## Graphical abstract

Prolonged high‐fat, high‐cholesterol feeding makes male mice more susceptible than females to obesity‐induced muscle atrophy, driven by disrupted intestinal homeostasis and mitochondrial dysfunction.

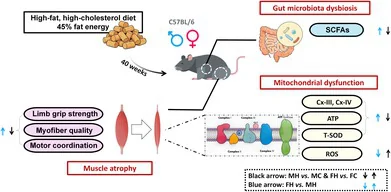

## 1 INTRODUCTION

The rise in obesity driven by high‐fat, high‐cholesterol (HFHC) diets has become a major global public health concern, often accompanied by a parallel increase in the incidence of obesity‐related chronic diseases. Notably, accumulating evidence indicates that obesity‐related chronic diseases exhibit marked sexual dimorphism under comparable HFHC dietary conditions (Goossens et al., 2021). Although obesity prevalence is higher in women than in men, men tend to develop obesity‐related skeletal muscle atrophy, hypertension, type 2 diabetes, coronary artery disease, heart failure, and stroke at an earlier age and at a lower BMI than women (Dakin et al., 2015; Luo et al., 2025; Moriggi et al., 2021). Beyond obesity as a common risk factor, sex differences in the incidence of obesity‐related chronic diseases also arise from the integrated effects of multiple sex‐specific pathophysiological factors, such as energy metabolism, gut microbiota, body fat distribution, and hormonal fluctuations.

Skeletal muscle atrophy is a common obesity‐induced chronic condition. It is characterized by a reduction in the cross‐sectional area (CSA) of skeletal muscle fiber, as well as a decline in muscle quality and quantity (Ren et al., 2025). Since skeletal muscle is one of the most energy‐demanding organs in the body, a chronic HFHC diet promotes insulin resistance, which in turn exacerbates skeletal muscle atrophy and dysfunction (Emerald et al., 2022). Specifically, long‐term HFHC feeding leads to continuous activation of stress‐related pathways, resulting in the inactivation of insulin signaling factors, mitochondrial dysfunction, and hyperglycemia. These changes trigger a cascade of metabolic remodeling and functional impairment (Castillo Í et al., 2025). In the context of obesity induced by prolonged HFHC diet consumption, muscle atrophy often coexists with impaired mitochondrial energy metabolism.

Although mitochondrial dysfunction is a hallmark of obesity‐induced muscle atrophy, the upstream triggers that initiate and sustain this bioenergetic failure remain incompletely understood. Emerging evidence suggests that gut microbiota dysbiosis may act as an upstream mediator of obesity‐related chronic diseases (Gandham et al., 2026; Sasidharan Pillai & Ashraf, 2026). Prolonged HFHC feeding disrupts the gut microbiota by reducing beneficial short‐chain fatty acid (SCFA)‐producing bacteria while increasing harmful Gram‐negative lipopolysaccharide‐producing bacteria (Du et al., 2025). This dysbiosis consequently diminishes SCFA synthesis, leading to decreased SCFA levels in both the gut and circulation. Of note, females maintain more stable microbial homeostasis under high‐fat diet stress than males, suggesting a sex‐dependent response to dietary challenges (Hases et al., 2023). Consistent with the concept of a gut‐muscle axis, circulating SCFAs activate AMPK and other energy sensors in myocytes, thereby regulating fatty acid metabolism and mitochondrial function to sustain skeletal muscle energy homeostasis (Xu & He, 2025). However, the sex‐specific mechanisms by which SCFAs modulate muscle metabolism remain poorly understood, underscoring the need for further investigation.

Prolonged HFHC feeding creates a state of chronic energy surplus, accompanied by gut microbiota dysbiosis and diminished SCFA production, ultimately leading to progressive functional deterioration of skeletal muscle mitochondria. This bioenergetic collapse is characterized by reduced activity of electron transport chain (ETC) complexes I and III, resulting in impaired adenosine triphosphate (ATP) synthesis and diminished cellular energy reserves (Sparks et al., 2005). Concomitantly, the dysfunctional ETC becomes a major source of reactive oxygen species (ROS) overproduction, which, together with a decline in total superoxide dismutase (T‐SOD) activity, overwhelms the endogenous antioxidant defense system (Zou et al., 2022). This persistent oxidative stress not only exacerbates mitochondrial damage but also disrupts cellular energy homeostasis. Consequently, the imbalance between elevated ROS generation and attenuated antioxidant capacity, combined with insufficient ATP production, creates a vicious cycle of bioenergetic failure that ultimately predisposes skeletal muscle to atrophy (Rasool et al., 2018). However, the mechanisms underlying sexual dimorphism in mitochondrial metabolic regulation within the context of obesity‐related muscle atrophy remain to be elucidated.

This study aimed to investigate the role of energy metabolic imbalance in the sexual dimorphism of obesity‐induced skeletal muscle atrophy and to delineate sex‐dependent differences in mitochondrial function and their association with the severity of muscle atrophy. To achieve this, male and female C57BL/6 mice were fed an HFHC diet for 40 weeks, modeling the long‐term dietary patterns associated with human metabolic dysfunction. By systematically comparing the two sexes with respect to physiological metabolism, gut microbiota composition, muscle function and quality, and mitochondrial performance, this study comprehensively evaluated the impact of sex on obesity‐related muscle atrophy. Its central objective was to elucidate sex differences in mitochondrial energy metabolism and how these differences relate to obesity‐induced muscle atrophy.

## 2 MATERIALS AND METHODS

### 2.1 Ethical approval

All animal procedures were approved by the Animal Ethics Committee of Guangdong Medical University (approval no: GDMU‐2023‐000064), and were conducted in strict accordance with relevant ethical guidelines for animal care and use.

### 2.2 Reagents

Assay kits for triglyceride (TG; E‐BC‐K261‐M), total cholesterol (TC; E‐BC‐K109‐M), high‐density lipoprotein cholesterol (HDL‐C; E‐BC‐K221‐M), and low‐density lipoprotein cholesterol (LDL‐C; E‐BC‐K205‐M) were procured from Elabscience Biotechnology Co., Ltd., Wuhan, China. Assay kits for aspartate aminotransferase (AST; C010‐2‐1), alanine aminotransferase (ALT; C009‐2‐1), total superoxide dismutase (T‐SOD; A001‐3), and reactive oxygen species (ROS; E004‐1‐1) were sourced from Nanjing Jiancheng Bioengineering Research Institute, Nanjing, China. The glucose assay kit (GLU; G1214W) was procured from Suzhou Grace Biotechnology Co., Ltd., Suzhou, China. The insulin ELISA kit (INS; BY‐EM220778) was acquired from Byabscience Biotechnology Co., Ltd., Nanjing, China. The adenosine triphosphate assay kit (ATP; S0026) was procured from Shanghai Beyotime Biotechnology Co., Ltd., Shanghai, China. Optimal cutting temperature compound (OCT; 4583) was procured from Sakura Finetek USA Inc., Torrance, CA, America. RNAiso Plus (9109), PrimeScript RT Reagent Kit (RR036A), and SYBR Premix Ex Taq II (RR820A) were sourced from Takara Biotechnology Co., Ltd., Tokyo, Japan. Primers for *Fbxo32*, *Trim63*, and the reference gene *Gapdh* were synthesized by Sangon Biotech Co., Ltd., Shanghai, China. Assay kits for mitochondrial complex I/NADH‐CoQ reductase activity (Cx‐I; BC0515), mitochondrial complex II/succinate‐coenzyme Q reductase activity (Cx‐II; BC3235), mitochondrial complex III/CoQ‐cytochrome C reductase activity (Cx‐III; BC3245), and mitochondrial complex IV/cytochrome C oxidase activity (Cx‐IV; 0945) were sourced from Beijing Solarbio Science & Technology Co., Ltd., Beijing, China. Methanol of UPLC/MS‐grade was obtained commercially from Tianjin Damao Chemical Reagent Factory, Tianjin, China.

### 2.3 Animals

C57BL/6 mice, aged 6 weeks, were obtained from the Guangdong Medical Experimental Animal Center (Foshan, China; certification 44007200133781). The mice were housed in a specific pathogen‐free (SPF) facility under controlled environmental conditions: temperature was maintained at 24°C ± 2°C, relative humidity at 50% ± 5%, with a 12‐h light/dark cycle. Food and water were provided ad libitum throughout the experimental period.

Sample size calculation was performed a priori using G*Power 3.1.9.7, for a two‐way ANOVA design with four experimental groups. The significance level was set at *α* = 0.05, and the statistical power was set at 0.80. Based on effect sizes reported in previous in vivo studies with a similar design, a Cohen's *f* of 0.60 was assumed, yielding a minimum required sample size of approximately 6.25 mice per group. To account for potential losses during routine animal husbandry and to enhance the reliability of experimental outcomes, the final sample size was set to 8 mice per group.

After 2 weeks of adaptive feeding, 32 mice were randomly divided into four groups: the male normal chow diet group (MC, *n* = 8), the female normal chow diet group (FC, *n* = 8), the male high‐fat and high‐cholesterol diet group (MH, *n* = 8), and the female high‐fat and high‐cholesterol diet group (FH, *n* = 8). The normal chow diet provided 3.7 kcal/g, with 12.1% of calories from fat, 65.9% from carbohydrates, and 21.9% from protein (SPF‐F02‐001, SiPeiFu, Beijing, China). The HFHC diet provided 20% kcal from protein, 35% kcal from carbohydrates, and 45% kcal from fat (D09100310, XiaoShuYouTai, Beijing) and was administered for 40 weeks to model the physiological state of middle‐aged women prior to the major decline in estrogen levels (Agrawal et al., 2025; Dutta & Sengupta, 2016), thereby minimizing the confounding effects of hormonal fluctuations on sex differences in muscle atrophy. All experimental diets were autoclaved to ensure sterility. Body weight and food intake of the mice were measured once a week for 40 weeks. Detailed compositions of the experimental diets are provided in Table S1.

During the final week of the animal experiment, behavioral tests were conducted, including the forelimb and four‐limb grip strength test using a grip strength meter (Haifuda Technology Co., Ltd., Beijing, China)., the rotarod tests using a rotarod apparatus (ZS Dichuang Technology Development Co., Ltd., Beijing, China), and the Y‐maze test and open field test using a video tracking system (Shanghai Xinruan Information Technology Co., Ltd., Shanghai, China). Prior to sample collection, mice were fasted for 12 h and then anesthetized via intraperitoneal injection of sodium pentobarbital (50 mg/kg). Blood was collected via eyeball enucleation. The blood samples were allowed to clot at room temperature for 2 h. Subsequently, the samples were centrifuged at 3000 × *g* at 4°C for 10 min, and the serum supernatant was carefully collected. Mice were euthanized by cervical dislocation. The gastrocnemius (GAS) muscles from both hindlimbs were completely dissected, weighed, and their weights recorded. The GAS index was calculated as (weight of GAS from both hindlimbs/body weight) × 100%. The collected serum, intestinal contents, and partial skeletal muscle tissues were immediately stored in liquid nitrogen, then transferred to a −80°C freezer for long‐term preservation until analysis.

### 2.4 Measurement of serum biochemical parameters

TG, TC, HDL‐C, LDL‐C, AST, ALT, fasting blood glucose (FBG), and fasting insulin (FINS) in serum were quantitatively determined by the colorimetric method in accordance with the instructions of the corresponding commercial assay kits. The homeostasis model assessment of insulin resistance (HOMA‐IR) was calculated from Equation (1) (Gao et al., 2021):
(1)

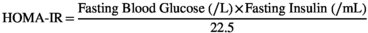




### 2.5 Histologic analysis

Partial GAS underwent tissue fixation in 4% paraformaldehyde, followed by paraffin embedding, serially sectioned at a thickness of 5 μm, and hematoxylin and eosin (H&E) staining. The histological processing was conducted by the Institute of Health and Medicine, Hefei Comprehensive National Science Center (Hefei, China). CaseViewer software (3DHISTECH Ltd., Budapest, Hungary) was used to evaluate pathomorphological changes and quantify muscle fiber CSA.

Partial GAS tissue was embedded in OCT medium, frozen on dry ice to form an embedding block, and then sectioned into 8 μm thick slices using a cryostat for immunofluorescence staining. The sections were fixed for 30 min in 4% paraformaldehyde at room temperature. After fixation, the sections were washed three times with phosphate‐buffered saline (PBS), 10 min per wash. In a dark environment, the sections were incubated with phalloidin for 60 min to label F‐actin, followed by three PBS washes of 10 min each. Nuclei were stained with DAPI. Immunofluorescence images were captured from sealed coverslips using a Leica TCS SP8 confocal microscope (equipped with an HC PL APO 63×/1.40 oil CS objective) and Leica Application Suite X 3.5.7 software. For quantification, eight mice per group were examined. A minimum of three randomly selected sections per mouse and three to five random fields of view per section were imaged. All acquired images were analyzed using ImageJ software, and mean values were calculated for each mouse for subsequent statistical analysis. Fluorescence intensity was also quantified using ImageJ software.

### 2.6 Quantitative real‐time PCR

Total RNA was extracted from the muscle tissues of each mouse group using RNAiso Plus, following the manufacturer's instructions. RNA concentration and purity were assessed on a NanoDrop 2000 spectrophotometer, and all samples were adjusted to a uniform RNA concentration before subsequent procedures. cDNA synthesis and quantitative real‐time PCR were carried out using the PrimeScript RT Reagent Kit and SYBR Premix Ex Taq II. cDNA synthesis was performed on a thermal cycler (Mastercycler® X40, Hamburg, Germany), and quantitative real‐time PCR was performed on an Applied Biosystems QuantStudio 5 Real‐Time PCR System (Thermo Fisher Scientific, USA). The amplification protocol included an initial 5 min denaturation step at 95°C, followed by 40 cycles of 5 s at 95°C and 30 s at 60°C, with a dissociation curve analysis at 60°C for 30 s. Relative mRNA expression levels of *Fbxo32* and *Trim63* were calculated using the 2^−ΔΔCT^ method, with *Gapdh* serving as the reference gene. All primer sequences are listed in Table S2.

### 2.7 Measurement of mitochondrial function and oxidative stress in skeletal muscle tissue

Skeletal muscle tissue was homogenized using a mechanical homogenizer in ice‐cold saline at a tissue‐to‐buffer ratio of 1:9 (w/v). The homogenate was then centrifuged at 11,000 × *g* for 15 min at 4°C. The resulting supernatants (tissue lysates) were aliquoted and used for biochemical analysis. The levels of mitochondrial complexes I‐IV, ROS, T‐SOD, ATP in skeletal muscle were quantitatively determined using colorimetric methods according to the instructions provided with the respective commercial assay kits. These values were normalized to the total protein concentration, which was measured using the bicinchoninic acid (BCA) assay.

### 2.8 Gut microbiota analysis

The collected colon contents were processed as follows: total genomic DNA was extracted from the samples using the CTAB/SDS method. DNA concentration and purity were evaluated by 1% agarose gel electrophoresis. Based on the measured concentration, DNA was diluted to 1 ng/μL with sterile water. Specific regions of the 16S rRNA were amplified with respective specific primers and barcodes. Subsequent data analysis was performed using the Majorbio Cloud platform.

### 2.9 Gas chromatography–mass spectrometric detection of SCFAs

Standardized procedures were used to prepare colon content and serum samples for SCFA analysis. Colon contents were homogenized with methanol at a ratio of 1∶4 (w/v), whereas serum samples were diluted with methanol at 1∶9 (v/v), followed by centrifugation at 12,000 × *g* for 15 min at 4°C. The SCFA profile—including acetic, propionic, butyric, and valeric acid—was determined by gas chromatography–mass spectrometry (GC–MS) using an Agilent 7890B/5977A instrument. Chromatographic conditions were as follows: DB‐FFAP column; helium carrier gas at 1.2 mL/min; 1 μL injection volume (split 10:1); oven temperature programmed from 60°C to 230°C at 15°C/min with a 3 min final hold; and inlet, ion source, and quadrupole temperatures set at 250°C, 230°C, and 150°C, respectively.

### 2.10 mRNA‐seq and non‐targeted transcriptomics analyses

GAS samples from mice in each group were processed by Shanghai Majorbio Bio‐pharm Technology Co., Ltd. for RNA extraction and quality control. Qualified RNA was then used to construct transcriptome libraries and perform sequencing. Differentially expressed genes (DEGs) were identified from the sequencing data, followed by functional enrichment analysis. Enrichment analysis of these DEGs was performed using the Gene Ontology (GO) and Kyoto Encyclopedia of Genes and Genomes (KEGG) databases to determine their distribution across biological processes, cellular components, molecular functions, and signaling pathways. Subsequently, key genes associated with mitochondria were screened, and gene expression levels were compared across the four groups to identify critical DEGs.

### 2.11 Statistical analysis

All experiments were performed independently at least three times, with each group consisting of 6–8 mice. Statistical analyses and graphical representations were performed using GraphPad Prism 9.5. Prior to analysis, data normality was assessed to justify the use of parametric tests. When the data met the assumptions of normality and homogeneity of variance, a two‐way analysis of variance (two‐way ANOVA) was conducted. When the diet × sex interaction was statistically significant, a simple effects analysis was performed, followed by Tukey's multiple comparison test for post hoc pairwise comparisons. When the data did not meet the assumptions of normality or homogeneity of variance, a Kruskal–Wallis test was performed, followed by Dunn's multiple comparison test for post hoc pairwise comparisons. Bioinformatic analysis of the gut microbiota was performed on the Majorbio Cloud platform (https://cloud.majorbio.com). Results are presented as the mean ± standard deviation, with statistical significance defined as *p* < 0.05.

## 3 RESULTS

### 3.1 HFHC diet increases body weight and disrupts glucose and lipid metabolism in both male and female mice

MC and FC mice were maintained on a standard chow diet for 40 consecutive weeks, while MH and FH mice received an HFHC diet for the same duration (Figure 1a). Compared to the MC group, the MH group showed an increasing trend in body weight starting at the 10th week of HFHC diet intervention. In contrast, female mice in the FH group exhibited an increasing trend in body weight compared to the FC group until the 20th week of the same dietary regimen. The final body weight of the MH group was 46% higher than that of the MC group, and the FH group was 34% higher than that of the FC group (Figure 1b). Analysis of cumulative body weight gain over the 40‐week experimental period revealed that both the MH and FH groups gained significantly more weight than their respective sex‐matched control groups (MC and FC). Body weight gain in the MH group was 234% higher than that in the MC group, while the FH group was 200% higher than that in the FC group. Furthermore, body weight gain in the MH group was 79% higher than that in the FH group, indicating a significant sexual dimorphism (Figure 1c). Regarding caloric intake, statistically significant differences were detected between the MC and MH groups and between the FC and FH groups, indicating that the observed body weight gain in the MH and FH groups was attributable to fat intake (Figure 1d).

**FIGURE 1:**
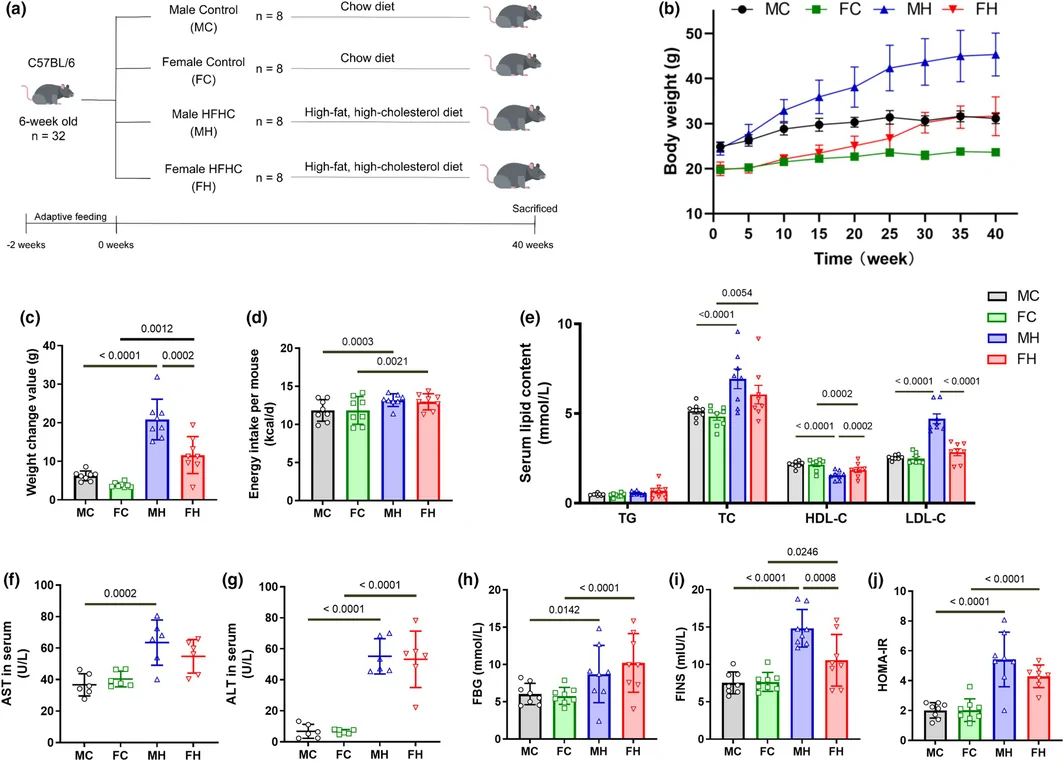
HFHC diet alters body weight and glucose‐lipid metabolism in both male and female mice. (a) Flowchart of the animal experiment. (b) Body weights. (c) Weight gain. (d) Daily caloric intake. (e) Serum lipid profiles. (f, g) Serum concentrations of AST and ALT (*n* = 6). (h) Fasting blood glucose. (i) Fasting insulin. (j) HOMA‐IR index. Results are expressed as mean ± SD from samples of six to eight mice per group.

Biochemical analyses of serum samples revealed that long‐term feeding of HFHC diet induced marked dysregulation of lipid metabolism in both sexes. This was evidenced by significantly elevated levels of TC, LDL‐C, AST, and ALT, along with a marked reduction in HDL‐C levels compared to their respective controls, and these changes were especially pronounced in males (Figure 1e–g). Notably, among these parameters, serum HDL‐C levels in the MH group were significantly lower than those in the FH group, while serum LDL‐C levels were significantly higher in the MH group than in the FH group. Additionally, compared with the FH group, the MH group showed increasing trends in TC and AST levels. FBG, FINS, and the HOMA‐IR index were all significantly elevated in both MH and FH groups relative to their respective controls, with the MH group showing significantly higher FINS levels than the FH group (Figure 1h–j). These findings confirm that a 40‐week HFHC diet induces systemic lipid and glucose metabolic disorders in both sexes, with more severe perturbations in males, indicating a sex‐specific susceptibility to HFHC‐induced metabolic dysfunction.

Sex and diet main effects and their interaction were further assessed by 2 × 2 factorial analysis, with full results in Table S3. Significant sex‐by‐diet interactions were observed for LDL‐C (*p* = 0.001) and FINS (*p* = 0.013). Consistent with the pairwise comparisons, the HFHC‐associated increases in LDL‐C and FINS were more pronounced in male mice. Among the indices without significant sex‐by‐diet interactions, significant diet effects were observed for TC (*p* = 0.001), HDL‐C (*p* < 0.001), AST (*p* = 0.001), ALT (*p* < 0.001), FBG (*p* = 0.003), and HOMA‐IR (*p* < 0.001).

### 3.2 HFHC diet induces more severe muscle atrophy and functional impairment in male mice than in females

Muscle function is a reliable indicator of atrophy severity. Grip strength was reduced in HFHC diet‐fed mice compared to sex‐matched control groups (e.g., MH vs. MC and FH vs. FC; Figure 2a,b). After adjusting for body weight, the adjusted mean forelimb grip strength in the MH group was 42.5% lower than that in the FH group. A similar trend was observed in the four‐limb grip strength measurements. Results from the rotarod test demonstrated that the exhaustive running time of the MH group was significantly shorter than that of both the MC and FH groups (Figure 2c). In the Y‐maze and open‐field tests, HFHC diet feeding caused a significant reduction in locomotor distance in the MH group, whereas no significant change was observed in the FH group (Figure 2d–f). Specifically, in the Y‐maze, HFHC diet feeding induced a significant decrease in both distance traveled and arm entries in the MH group compared to the MC group. In contrast, the FH group showed decreases in both parameters relative to the FC group, but these did not reach statistical significance. Furthermore, the MH group exhibited decreasing trends in both distance traveled and arm entries compared to the FH group. The open field test results showed that, compared to the MC group, the MH group exhibited a decreasing trend, whereas no significant change was observed between the FC and FH groups. These results indicate that the HFHC diet leads to a marked decline in motor coordination and locomotor activity in male mice, while female mice are less susceptible to the deleterious effects of the HFHC diet.

**FIGURE 2:**
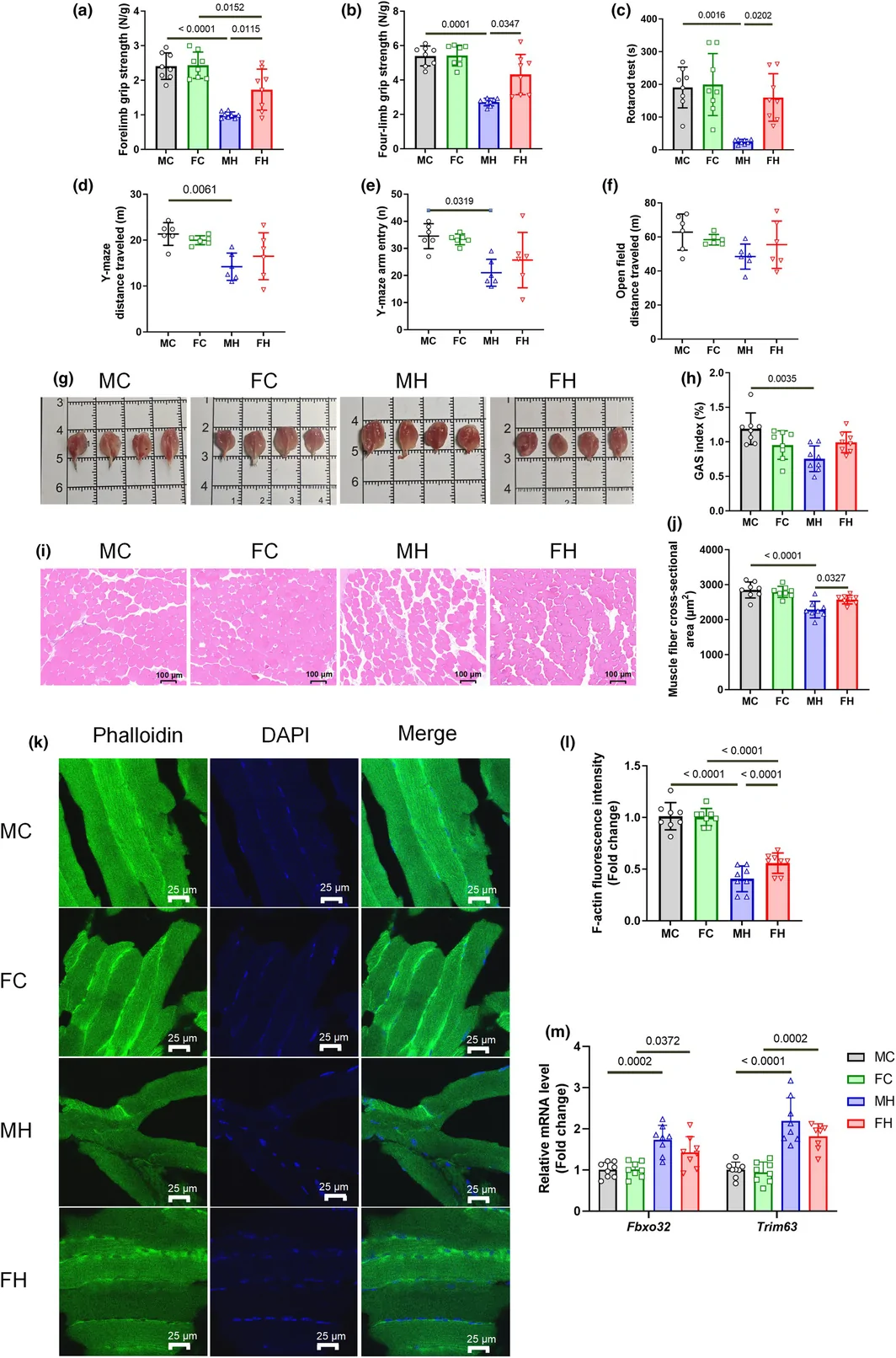
Sexual dimorphism is evident in HFHC‐induced muscle atrophy and functional impairment, with male mice exhibiting more severe deficits in muscle quality, strength, and function compared to females. (a) Forelimb tension. (b) Limb tension. (c) Mean time on the rotarod test. (d) Total distance traveled in the Y‐maze. (e) Total number of arm entries in the Y‐maze. (f) Distance traveled in the open field test. (g) Representative images of the GAS (*n* = 4). (h) GAS index. (i) H&E staining (scale bar = 100 μm). (j) Measurement of myofiber CSA. (k) Phalloidin staining (scale bar = 25 μm). (l) Quantification of phalloidin intensity. (m) *Fbxo32* and *Trim63* mRNA expression levels. Results are expressed as mean ± SD from samples of four to eight mice per group.

Skeletal muscle atrophy is characterized by a decline in both the quality and quantity of muscle fibers. Compared to the MC and FC groups, the MH and FH groups showed reduced GAS muscle mass, with a more pronounced decline in the MH group (Figure 2g,h). GAS mass was significantly lower in the MH group than in the MC group, and tended to be lower than in the FH group. Analysis of GAS fiber CSA on HE‐stained sections (Figure 2i,j) revealed a significant reduction in the MH group. The GAS fiber CSA in the MH group was 11.2% lower than that in the FH group. GAS F‐actin staining visually revealed a lower degree of actin alignment in the MH group, whereas the FH group appeared similar to the FC group (Figure 2k). Regarding F‐actin fluorescence intensity, both the MH and FH groups showed decreased intensity following HFHC feeding (Figure 2l). Compared to the MC group, the MH group showed a 59.9% reduction in F‐actin fluorescence intensity, while the FH group demonstrated a 44.4% decrease relative to the FC group. Additionally, the MH group displayed a 27.2% lower F‐actin fluorescence intensity than the FH group, a difference that was statistically significant. Concurrently, the expression of atrophy‐related genes was altered. The MH group exhibited a significant increase in *Fbxo32* and *Trim63* mRNA expression compared to the MC group. Similarly, the FH group showed elevated expression of these genes relative to the FC group. Although the MH group tended to have higher *Fbxo32* and *Trim63* expression than the FH group, this difference did not reach statistical significance (Figure 2m).

Sex and diet main effects and their interaction were further assessed by 2 × 2 factorial analysis, with full results in Table S3. Significant sex‐by‐diet interactions were observed for GAS index (*p* = 0.002), muscle fiber CSA (*p* = 0.019), and F‐actin fluorescence intensity (*p* = 0.003). Among the indices without significant sex‐by‐diet interactions, significant diet effects were observed for forelimb grip strength (*p* < 0.001), four‐limb grip strength (*p* < 0.001), rotarod test (*p* = 0.003), Y‐maze distance traveled (*p* = 0.001), Y‐maze arm entry (*p* < 0.001), open field distance traveled (*p* = 0.039), *Fbxo32* (*p* < 0.001), and *Trim63* (*p* < 0.001).

### 3.3 HFHC diet induces more severe skeletal muscle mitochondrial dysfunction and oxidative stress in male mice than in females

As illustrated in Figure 3a, the activity of mitochondrial Cx‐I in both the MH and FH groups was significantly lower than that in their respective control groups. Regarding Cx‐III activity, both MH and FH groups displayed a substantial decline compared to their corresponding controls, with a more pronounced decrease observed in males. The MH group showed a 24.5% lower activity than the FH group. For Cx‐IV, the MH group showed a marked reduction in enzyme activity compared to the MC group, with activity 60.1% lower than that of the FH group.

**FIGURE 3:**
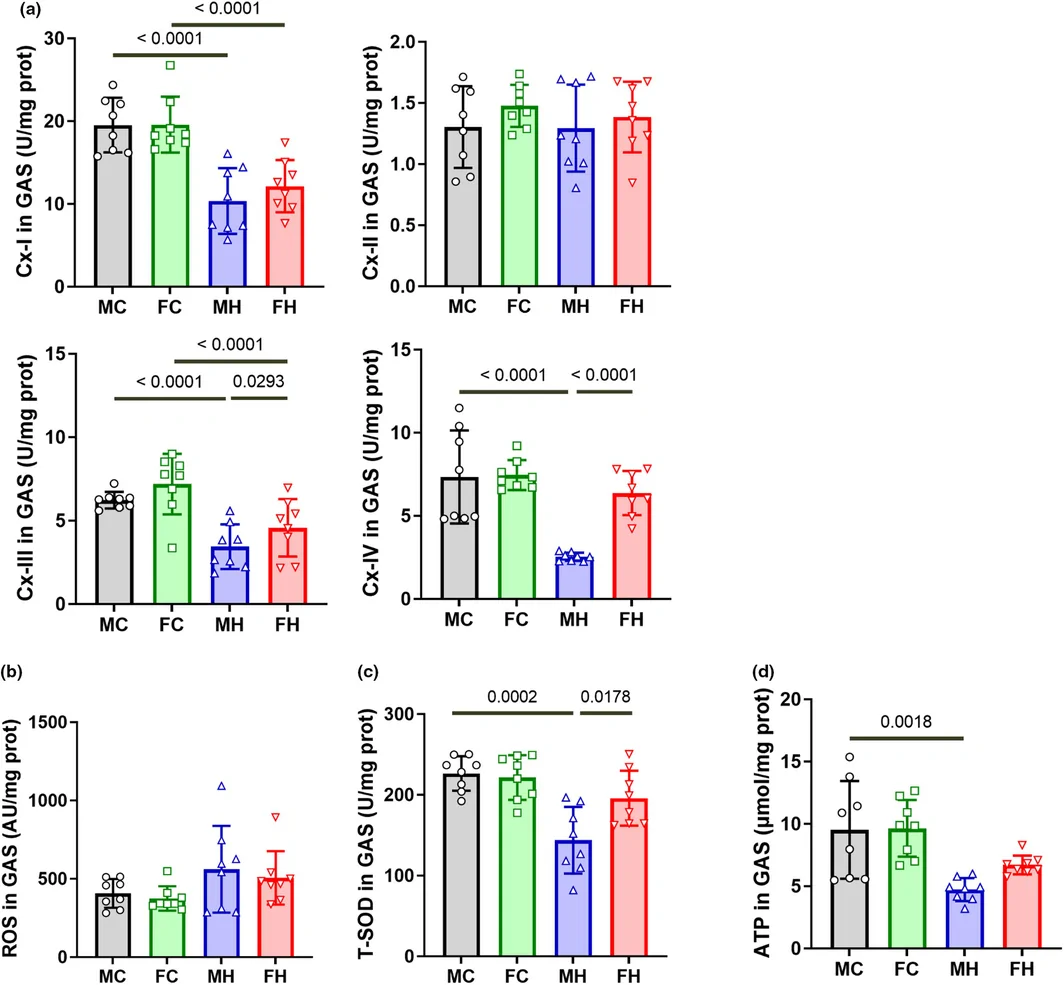
HFHC diets impaired mitochondrial oxidative phosphorylation and elevated oxidative stress in mice, with these detrimental effects being more pronounced in males. (a) Activity levels of complexes I‐IV in GAS. (b) ROS levels in GAS. (c) T‐SOD activity in GAS. (d) ATP levels in GAS. Results are expressed as mean ± SD from samples of eight mice per group.

Elevated levels of ROS indicate dysfunction in the mitochondrial ETC. The results presented in Figure 3b indicate an increasing trend in ROS content in the GAS of both the MH and FH groups. Based on these findings, it is speculated that the HFHC diet may induce oxidative stress in mouse muscle tissue, with no observed sex differences between male (MH) and female (FH) mice.

T‐SOD is a key component of the mitochondrial antioxidant defense system, primarily responsible for scavenging superoxide anion radicals that are harmful to cells. Compared to the MC group, the MH group showed a significant decrease in T‐SOD levels. The FH group exhibited a mild, non‐significant decrease relative to the FC group. Notably, the T‐SOD level in the MH group was 27.0% lower than that in the FH group (Figure 3c).

ATP levels directly reflect the efficiency of mitochondrial oxidative phosphorylation. Biochemical analysis indicated that ATP content in the MH group was significantly lower than that in the MC group, whereas only a downward trend was observed in the FH group relative to the FC group. Additionally, the MH group tended to have lower ATP levels than the FH group (Figure 3d).

Collectively, these findings confirm that HFHC diet induces oxidative stress in the muscle mitochondria of mice and disrupts normal mitochondrial energy metabolism. Furthermore, the degree of mitochondrial dysfunction in female mice (FH group) was less severe than in male mice (MH group), and the mitochondrial antioxidant defense system in the FH group was more stable compared to that in the MH group, highlighting a distinct sex difference in susceptibility to HFHC diet‐induced mitochondrial impairment.

Sex and diet main effects and their interaction were further assessed by 2 × 2 factorial analysis, with full results in Table S3. Significant sex‐by‐diet interactions were observed for Cx‐IV (*p* = 0.001), and T‐SOD (*p* = 0.017). Among the indices without significant sex‐by‐diet interactions, significant diet effects were observed for Cx‐I (*p* < 0.001), Cx‐III (*p* < 0.001), and ATP (*p* = 0.001).

### 3.4 HFHC diet reshapes gut microbiota composition in a sex‐dependent manner, with females exhibiting an enhanced homeostatic capacity

The Venn diagram showed that the HFHC diet reduced gut microbiota abundance in both the MH and FH groups (Figure 4a). The number of differential taxa between the MH and MC groups reached 50, while that between the FH and FC groups was 37. The number of differential taxa in the MH group was 22 fewer than that in the FH group. The results of the Rank‐Abundance curves indicated that the MH group had the shortest curve, representing the lowest species richness (Figure 4b). In contrast, the curves of the MC and FC groups were relatively smooth, indicating the highest species evenness among the four groups. Three‐dimensional principal coordinate analysis (3D‐PCoA) based on β‐diversity metrics revealed distinct clustering of gut microbial communities among the four experimental groups (Figure 4c). Figure 4d–f present the Sobs, Chao, and Shannon indices of α‐diversity. The Sobs and Chao indices demonstrated that species richness in the MH and FH groups decreased significantly compared with the MC and FC groups, respectively. As a comprehensive index reflecting species richness and evenness, the Shannon index showed a downward trend in both the MH and FH groups. Non‐metric multidimensional scaling analysis revealed significant differences in gut microbiota composition between the MH and MC groups, as well as between the FH and FC groups. Moreover, the gut microbiota composition of the FH group was closer to that of the control group than that of the MH group.

**FIGURE 4:**
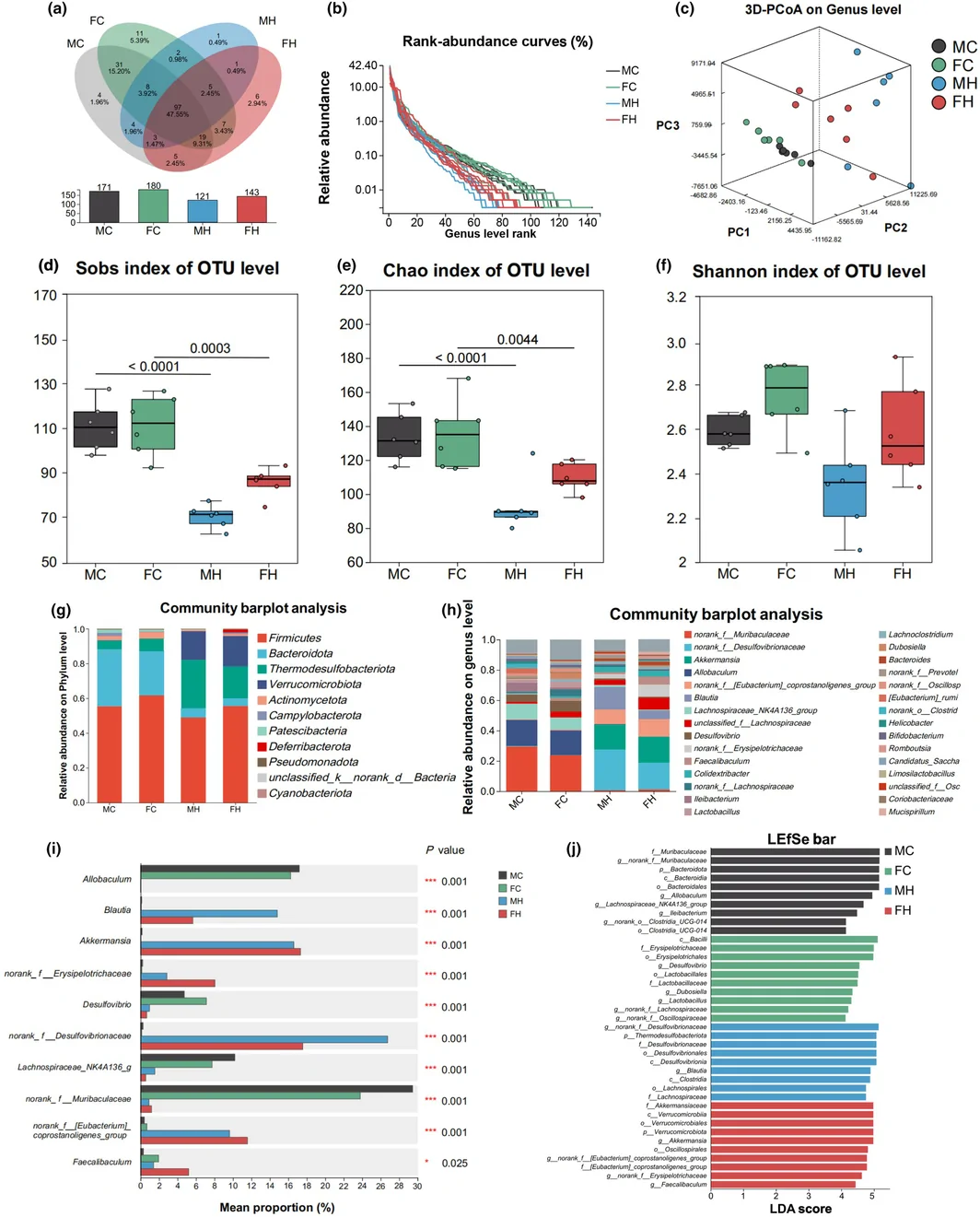
Male mice exhibit greater susceptibility to HFHC‐induced gut microbiota dysbiosis, as demonstrated by more pronounced compositional disruptions compared to females. (a) Venn diagram of operational taxonomic units (OTUs) in the MC, FC, MH, and FH groups. (b) Rank‐abundance curves: The length of the horizontal axis represents species richness, while the smoothness of the curves indicates species evenness. (c) Beta‐diversity of gut microbiota based on non‐metric multidimensional scaling analysis. (d) Alpha‐diversity of gut microbiota assessed by Sobs indices. (e) Chao indices. (f) Shannon indices. (g) Relative abundance of gut microbiota at the phylum level. (h) Relative abundance of gut microbiota at the genus level. (i) Differences in gut bacteria among the four groups. (j) Dominant gut microbiota among the four groups. Results are expressed as mean ± SD from samples of six mice per group.

At the phylum level, the HFHC diet caused a marked decrease in the abundance of *Bacteroidota*, while the levels of *Thermodesulfobacteriota* and *Verrucomicrobiota* increased (Figure 4g). At the genus level, several potentially harmful bacteria, such as *norank_f_Desulfovibrionaceae, Akkermansia, norank_f_[Eubacterium]_coprostanoligenes_group, Blautia, unclassified_f_Lachnospiraceae, norank_f_Erysipelotrichaceae*, increased. In contrast, beneficial bacteria, including *norank_f_Muribaculaceae, Allobaculum, Lachnospiraceae_NK4A136_group, Desulfovibrio*, and *Ileibacterium*, decreased in HFHC‐fed mice (Figure 4h). Among these genera, the abundance of *Blautia* was higher in the MH group than in the FH group. Conversely, the levels of *norank_f_Erysipelotrichaceae* and *unclassified_f_Lachnospiraceae* were higher in the FH group than in the MH group.

The LEfSe bar plots showed the top 10 differentially abundant gut microbiota taxa among the four groups (Figure 4i,j). Beneficial bacteria in the MH group included *o_Lachnospirales, f_Lachnospiraceae*, and *g_Blautia*, while those in the FH group comprised *c_Bacilli, o_Erysipelotrichales, f_Erysipelotrichaceae*, and *g_norank_f_Erysipelotrichaceae*.

### 3.5 HFHC diet induces a greater reduction in SCFA production in male mice than in females

Prolonged consumption of an HFHC diet induces gut microbial dysbiosis, characterized by a depletion of beneficial bacteria and an enrichment of pathogenic species. This dysbiosis impairs fermentative function and markedly suppresses SCFA production. As expected, HFHC diet feeding significantly decreased total SCFA levels in both serum and colonic contents of male and female mice. Notably, a significant difference in colonic total SCFAs was observed between the MH and FH groups (Figure 5a,b). Specifically, propionic acid levels in both serum and colonic contents were relatively lower in the FH and MH groups than in their respective controls, with a significant difference between the MH and MC groups and a downward trend in the MH group relative to the FH group. Butyric acid levels in serum were significantly decreased in the MH group compared with the MC group and showed a decreasing trend in the FH group compared with the FC group. Moreover, the MH group exhibited a 24.69% lower serum butyric acid level than the FH group. In colonic contents, butyric acid levels were significantly decreased in the MH group compared with the MC group, whereas only a decreasing trend was observed in the FH group relative to the FC group. The MH group also showed a 50.00% lower level than the FH group, and this difference was statistically significant. For acetate and valerate in both serum and colonic contents, levels in the MH group were significantly lower than those in the MC group, whereas the FH group showed only a slight reduction compared with the FC group; this reduction was less pronounced than that observed in male mice.

**FIGURE 5:**
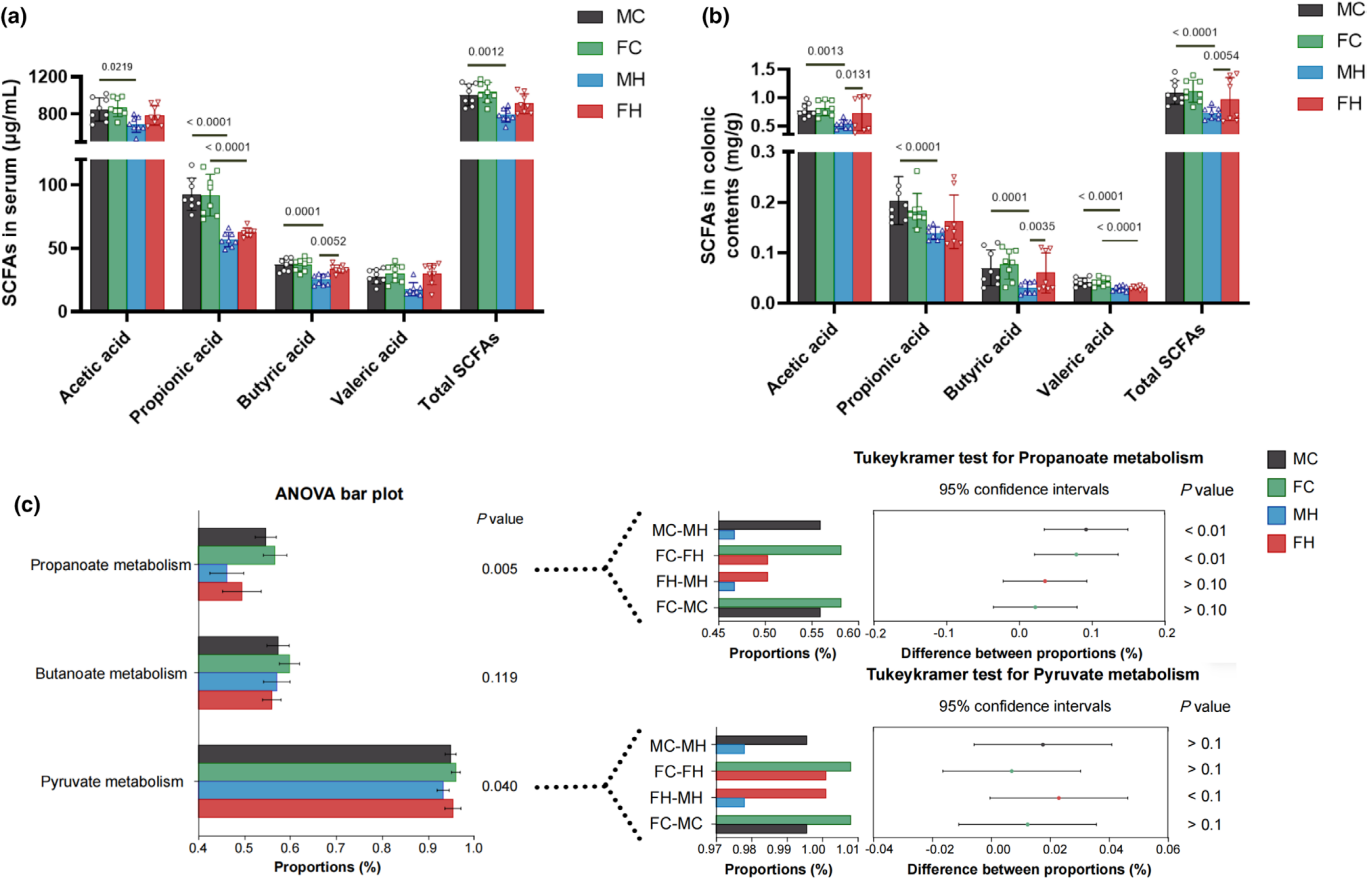
HFHC diet alters SCFA profiles in male and female mice. (a) Concentrations of SCFAs in serum across the four groups. (b) Concentrations of SCFAs in colonic contents across the four groups. (c) Differential abundance of SCFA metabolism‐related pathways predicted by PICRUSt2 (*n* = 6). Results are expressed as mean ± SD from samples of six to eight mice per group.

PICRUSt2 functional prediction further supported the finding that male mice have a reduced capacity to maintain intestinal homeostasis compared to females (Figure 5c). The results showed that propanoate metabolism was significantly lower in both the MH and FH groups compared to their respective controls (MC and FC), and it also exhibited a decreasing trend in the MH group relative to the FH group. Although no significant differences were observed, butanoate metabolism showed a downward trend in both the MH and FH groups. Similarly, pyruvate metabolism showed a decreasing trend in both MH and FH groups compared to their corresponding controls, as well as in the MH group relative to the FH group.

Based on the quantification of SCFAs and functional prediction results, the authors speculate that the HFHC diet inhibits SCFA production by disrupting gut microbiota homeostasis. This mechanism is associated with the downregulation of microbial SCFA biosynthesis.

Sex and diet main effects and their interaction were further assessed by 2 × 2 factorial analysis, with full results in Table S3. Significant sex‐by‐diet interactions were observed for butyric acid in serum (*p* = 0.011), valeric acid in serum (*p* = 0.048), and propionic acid in colonic contents (*p* = 0.036). Among the indices without significant sex‐by‐diet interactions, significant diet effects were observed for acetic acid in serum (*p* = 0.002), propionic acid in serum (*p* < 0.001), total SCFAs in serum (*p* < 0.001), acetic acid in colonic contents (*p* = 0.018), butyric acid in colonic contents (*p* = 0.007), valeric acid in colonic contents (*p* < 0.001), and total SCFAs in colonic contents (*p* = 0.005).

### 3.6 Effects of HFHC diet on the transcriptomic profiles of skeletal muscle: Emphasis on differentially expressed genes related to mitochondrial function

Transcriptomic analysis was conducted to identify potential novel genes associated with HFHC‐induced muscle atrophy. Compared to the MC group, the MH group exhibited 943 upregulated and 531 downregulated genes. Similarly, relative to the FC group, the FH group exhibited 687 upregulated and 662 downregulated genes. When comparing the FH group to the MH group, 236 genes were upregulated and 151 were downregulated (Figure S1a,b). A Venn diagram was used to compare the results of these three pairwise comparisons (Figure S1c), revealing 30 common differentially expressed genes (DEGs) at the intersection. Principal component analysis (PCA) demonstrated a clear separation between samples from different groups (Figure S1d). GO and KEGG pathway enrichment analyses were conducted on the DEGs. GO functional enrichment analysis encompassed three categories: biological processes (BP), molecular functions (MF), and cellular components (CC). GO terms with more than two differentially expressed genes in the BP, CC, and MF categories were selected. The top ten terms in each category were ranked by their –log_10_ *p*‐values in descending order, and the results for the top ten significantly enriched terms in each category are presented in Figure S1e–j. The results showed that muscle atrophy in male mice was primarily influenced by oxidative phosphorylation and energy metabolism, whereas female mice were more affected by the MAPK, FoxO, PI3K, and TNF signaling pathways involved in the regulation of protein metabolism and oxidative stress.

To further investigate the biological characteristics and potential mechanisms underlying sex differences in muscle atrophy, GSEA was performed on the differentially expressed genes between the MH group and FH group (Figure 6a). The results revealed that the differences in skeletal muscle atrophy between males and females were mainly concentrated in five areas: the tricarboxylic acid cycle, ETC, ATP synthase and proton transport, mitochondrial complex assembly and protein synthesis, mitochondrial structure and related processes. Subsequently, three representative genes from these pathways were selected for quantitative analysis of their expression levels (Figure 6b). The results revealed that, among genes related to mitochondrial structure and energy metabolism, the MH group exhibited decreased expression levels compared with the MC group, whereas the FH group showed negligible changes relative to the FC group. Moreover, the expression levels of mitochondrial genes such as *Sdhb*, *Ndufa11*, *Atp5me*, *Sdhaf4*, and especially *Coq10b* were higher in the FH group than those in the MH group, suggesting a more intact mitochondrial oxidative phosphorylation function in the females.

**FIGURE 6:**
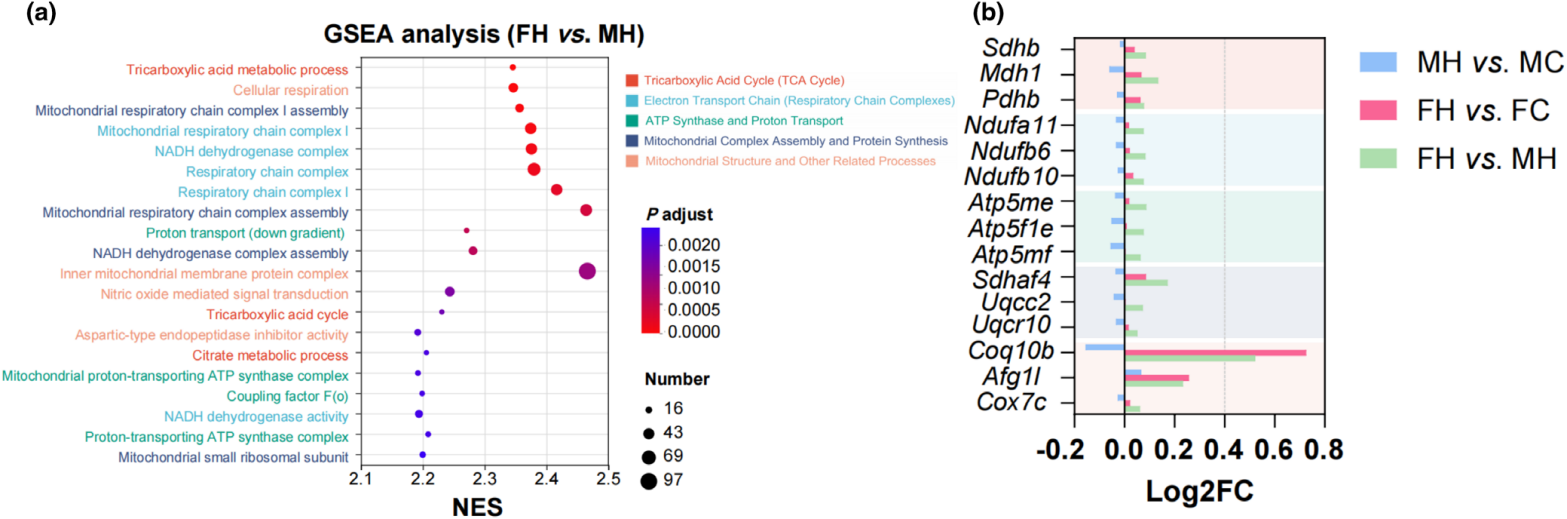
Quantitative analysis of DEGs in the GAS of the MH and FH groups of mice. (a) GSEA of DEGs comparing the FH group to the MH group. (b) Quantification of mitochondrial and energy metabolism‐related DEGs. Results are presented on the x‐axis as Normalized Enrichment Score (NES) and Log_2_FC, with data derived from samples of five mice per group.

### 3.7 Interaction among gut microbiota, SCFAs, and skeletal muscle atrophy

Spearman correlation analysis was performed to evaluate the relationships among gut microbiota, SCFAs, and muscle atrophy‐related parameters. Beneficial genera, including *norank_f_Muribaculaceae*, *Allobaculum*, *Lachnospiraceae_NK4A136_group*, *Desulfovibrio*, and *Lactobacillus*, showed positive correlations with both muscle quality and SCFA levels. In contrast, potentially harmful taxa such as *Akkermansia* and *Blautia* showed inverse associations (Figure 7a,b). Among SCFAs, most—except serum valeric acid—were correlated with bacterial taxa, particularly propionic and butyric acids.

**FIGURE 7:**
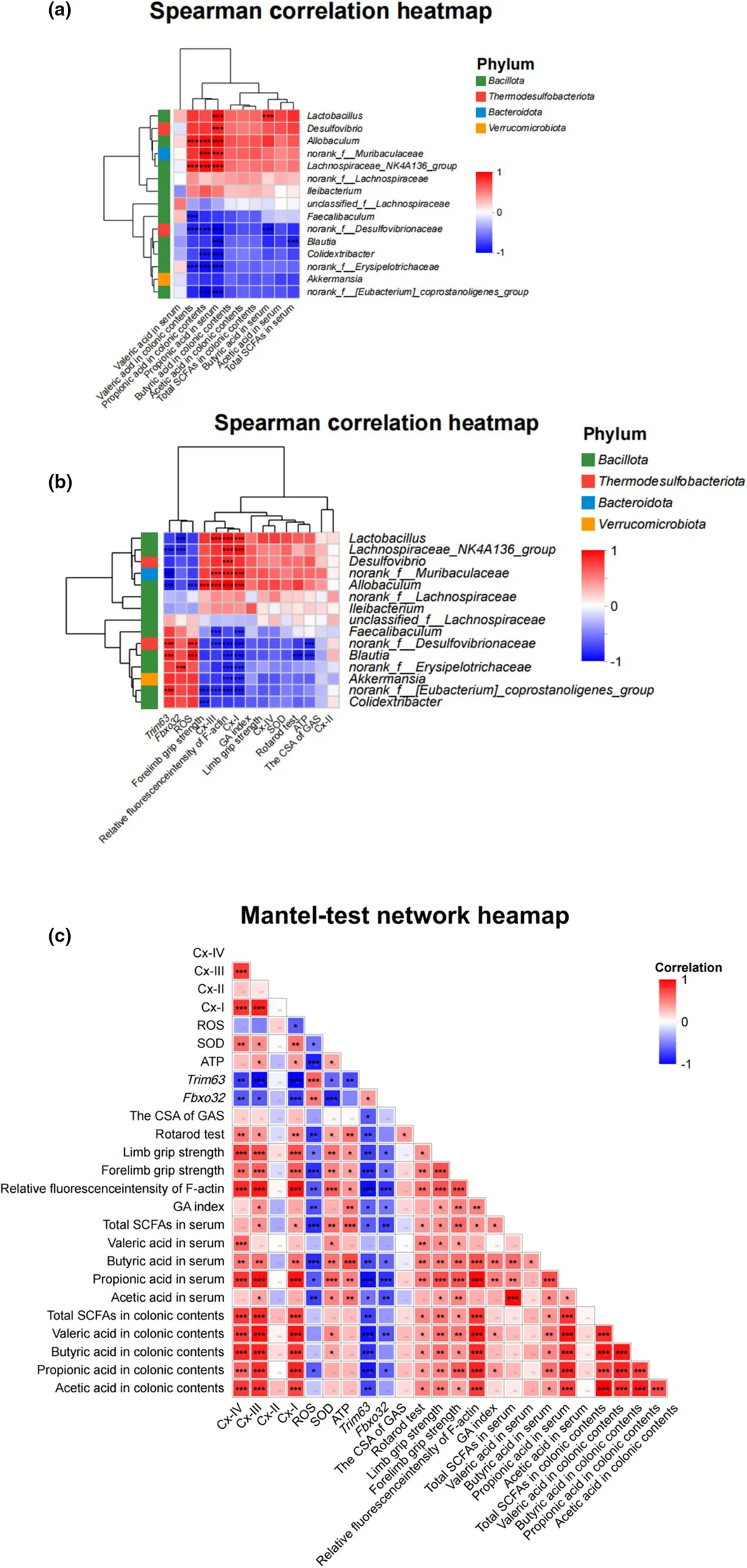
The relationship between gut microbiota, SCFAs, and muscle atrophy. (a) Heatmap visualizing Spearman correlations between gut microbiota and muscle phenotypic indicators, mitochondrial bioenergetics, oxidative stress markers, and atrophy‐related proteins (*n* = 6). (b) Correlation heatmap analysis of gut microbiota and SCFAs (*n* = 6). (c) Heatmap depicting correlations among muscle atrophy‐related parameters. In the heatmaps, red indicates positive correlations, while blue indicates negative correlations. Darker colors represent stronger correlations, and asterisks denote significance levels. Results are expressed as mean ± SD from samples of six to eight mice per group. *p < 0.05, **p < 0.01, ***p < 0.001.

Correlation analysis also revealed strong positive associations between mitochondrial function parameters (Cx‐I, Cx‐III, Cx‐IV, ATP) and muscle quality indicators, whereas oxidative stress markers showed positive correlations with atrophy‐related genes (Figure 7c). Collectively, these findings support the hypothesis that gut microbiota composition is linked to both SCFA production and the progression of muscle atrophy, although further mechanistic studies are required to establish causality.

## 4 DISCUSSION

In this study, male and female C57BL/6 mice were fed an HFHC diet for 40 weeks to model obesity and investigate the role of mitochondrial energy metabolic imbalance in the sexual dimorphism of obesity‐induced muscle atrophy. This long‐term feeding regimen better mimics the gradual development of metabolic diseases compared to short‐term feeding. The results showed that prolonged HFHC feeding disrupted gut microbiota and lipid homeostasis, induced mitochondrial dysfunction, and impaired energy uptake in skeletal muscle, ultimately exacerbating muscle atrophy. Although HFHC feeding is known to disrupt gut microbiota (Du et al., 2025), this study revealed that, despite similar increases in pathogenic bacteria in both sexes, females exhibited a smaller loss of beneficial bacteria and uniquely displayed compensatory taxa linked to microbiota restoration. These responses were absent in males, contributing to more stable levels of gut microbial metabolites (e.g., propionate and butyrate) and thus more stable internal homeostasis in females.

Recent evidence demonstrates that diets high in fat content can induce muscle atrophy and other obesity‐related chronic diseases in mice, with clear sex differences in overall health status, manifested as poorer outcomes in males. This sex difference is characterized by disturbances in glucose and lipid metabolism, alterations in gut microbiota composition, and exacerbated oxidative stress markers in male mice. Obesity‐induced muscle atrophy is marked by significant reductions in muscle mass, diminished muscle fiber CSA, and impaired muscle function. Following long‐term HFHC diet feeding, both male and female mice exhibited progressive body weight gain relative to standard‐diet controls, accompanied by reduced muscle strength, decreased muscle fiber CSA, and lower relative GAS weight. Notably, HFHC‐fed male mice showed greater declines in these indicators than HFHC‐fed females. These findings are consistent with previous studies showing that males are more susceptible to obesity‐induced muscle atrophy under high‐fat diets. However, daily energy intake was comparable between male and female mice fed the HFHC diet, indicating that sex differences in lipid metabolism and muscle quality induced by the HFHC diet were not attributable to differences in energy consumption. Despite equivalent caloric intake, male mice exhibited earlier onset, faster progression, and greater magnitude of body weight gain. Moreover, male mice showed significantly greater lipid metabolic dysregulation and insulin resistance than females, along with reduced gut microbial diversity, increased abundance of pathogenic bacteria, and decreased abundance of beneficial bacteria. Collectively, these data indicate that long‐term HFHC feeding induces a more pronounced metabolic imbalance in male mice.

Metabolic imbalance is a key factor driving obesity‐induced muscle atrophy. A dietary pattern with a high proportion of energy derived from fat promotes skeletal muscle atrophy through multiple pathways, including protein metabolic imbalance, insulin resistance, chronic low‐grade inflammation, and mitochondrial energy metabolic dysfunction, particularly impaired mitochondrial oxidative phosphorylation. The vulnerability of skeletal muscle to energy metabolic imbalance arises because normal muscle contractile function and the balance between protein synthesis and degradation critically depend on energy homeostasis. Among these factors, the functional integrity of the mitochondrial ETC plays a central role. In this study, both male and female mice fed the HFHC diet exhibit impaired oxidative phosphorylation in skeletal muscle, as evidenced by significantly reduced activities of complexes Cx‐I, Cx‐III, and Cx‐IV. This disruption impairs the formation of the proton gradient across the inner mitochondrial membrane, leading to insufficient ATP production. This energy deficit not only limits normal skeletal muscle contraction but also triggers excessive ROS production. Excessive ROS attack mitochondrial membrane lipids and proteins, further damaging mitochondrial function. Damaged mitochondria then produce even more ROS, worsening oxidative damage and creating a vicious cycle of “ROS‐induced mitochondrial damage”.

After HFHC feeding, male mice showed a more severe homeostatic imbalance in mitochondrial function compared to females, mainly reflected by decreased activities of Cx‐III and Cx‐IV. Since Cx‐I, Cx‐III, and Cx‐IV are responsible for pumping protons and transferring electrons within the mitochondrial ETC. They actively pump protons against their concentration gradient into the intermembrane space while transferring electrons along two main pathways: “Cx‐I → Cx‐III → Cx‐IV” and “Cx‐II → Cx‐III → Cx‐IV”. This process establishes the proton gradient necessary for ATP production, and the directed electron transfer provides the energy required to reduce oxygen to water. Given the critical roles of Cx‐III and Cx‐IV in establishing the proton gradient and sustaining ATP synthesis, the significant reduction in their activities in male mice may represent a key factor underlying the sexual dimorphism observed in obesity‐induced muscle atrophy. These complexes function as primary proton pumps and serve as convergence points for the two electron transfer pathways. Thus, their reduced activities in male mice impair proton gradient formation and ATP production, which may account for the decline in muscle function observed in males. Moreover, since electron transfer drives mitochondrial oxygen consumption, decreased activities of these complexes slow the entire ETC, leading to insufficient oxygen utilization and ultimately respiratory inhibition within mitochondria. Meanwhile, the reduced electron transfer rate at Cx‐III causes electron accumulation, which further triggers excessive ROS production. Therefore, mitochondria are subjected to multiple adverse effects, including impaired energy production, ETC inhibition, and oxidative stress. Overall, male mice fed the HFHC diet exhibited significantly greater ETC impairment than females, further compromising normal mitochondrial energy production.

Impaired mitochondrial oxidative phosphorylation is influenced by multiple upstream factors, including systemic metabolic disturbances, chronic low‐grade inflammation, as well as altered sex hormone signaling, such as estrogen. It is important to acknowledge that estrogen stability is, to some extent, beneficial for maintaining mitochondrial function (Pellegrino et al., 2022). Meanwhile, studies have shown that in human females, the precipitous decline in estrogen after menopause is associated with a greatly increased incidence of muscle wasting (Menzies et al., 2026). Estrogen may therefore play a role in preserving muscle quality in female mice under HFHC diet. In addition to the influence of sex hormones, impaired mitochondrial oxidative phosphorylation also results from systemic metabolic dysregulation. As a component of this dysregulation, gut microbiota dysbiosis is one of the most directly affected targets of the HFHC diet. Analysis of gut microbiota reveals that female mice maintain a more stable intestinal environment, which may contribute to their resilience against impaired mitochondrial oxidative phosphorylation caused by long‐term HFHC feeding. Conversely, male mice fed the HFHC diet exhibit more pronounced ETC damage. Sex‐based variations in gut microbial composition may partly explain the sexual dimorphism observed in oxidative phosphorylation within skeletal muscle mitochondria. Meanwhile, previous studies have demonstrated that, under identical HFHC dietary conditions, female mice exhibit greater resilience than males in maintaining gut microbiota homeostasis (Hases et al., 2023).

The present study further revealed that HFHC diet feeding markedly reduced the abundance of SCFA‐producing beneficial bacterial taxa, including *norank_f_Muribaculaceae*, *Allobaculum*, *Lachnospiraceae_NK4A136_group* and *Desulfovibrio*, in both male and female mice, leading to decreased SCFA levels in the gut (Li et al., 2025; Zheng et al., 2021; Zhu et al., 2024). According to Xie et al., SCFAs alleviate obesity‐induced muscle atrophy by restoring gut microbial homeostasis and inhibiting ubiquitin‐proteasome system (UPS)‐dependent protein degradation (Xie et al., 2026). Concurrently, HFHC diets markedly increased the gut abundance of atrophy‐associated bacterial taxa‐*norank_f_Desulfovibrionaceae* and *Blautia*‐in both male and female mice, with a more pronounced elevation observed in males (Sakaguchi et al., 2025; Wang et al., 2022). In addition, certain bacterial taxa have been reported to promote muscle repair and improve muscle function, including *Akkermansia* and *norank_f_Erysipelotrichaceae*. Following HFHC diet feeding, the abundance of both taxa was significantly higher in mice of both sexes compared to their respective controls (Barberá et al., 2026; Kang et al., 2024; Zhang et al., 2024). Interestingly, female mice fed the HFHC diet exhibited significantly higher abundances of these beneficial taxa than their male counterparts. HFHC diet feeding accelerates muscle atrophy through depletion of SCFA‐producing bacteria, a mechanism that does not differ between sexes. Notably, females showed less enrichment of harmful bacteria and greater compensatory increases in muscle‐repairing microbiota than males under metabolic challenge. The gut microbiota of female mice is inherently poised to restrain the expansion of deleterious bacterial taxa while promoting the enrichment of repair‐mediating species, a compositional bias that may, relative to males, enhance internal homeostasis, ETC homeostasis, and resistance to muscle atrophy.

The effects of the gut microbiota extend beyond those described above. In both male and female mice fed the HFHC diet, gut dysbiosis led to a significant reduction in SCFA production, including propionic acid and butyric acid. Propionic acid, a key metabolite of gut microbial fermentation, is recognized as an important signaling molecule that influences host metabolism. Recent evidence indicates that propionic acid exerts protective effects on mitochondrial function and cellular energy homeostasis, partly through its interaction with the G protein‐coupled receptors GPR41 and GPR43, which are expressed in skeletal muscle (Wang et al., 2025). Based on these findings, it is plausible that the decreased propionic acid levels observed in HFHC‐fed mice contribute to impaired mitochondrial respiratory chain activity (complexes I, III, and IV), reduced ATP synthesis, and elevated ROS production detected in this study. Notably, the more pronounced depletion of propionic acid in male mice correlated with greater severity of mitochondrial dysfunction and oxidative stress, suggesting that propionic acid reduction may be a key factor linking gut microbiota dysbiosis to sex‐specific muscle atrophy. However, direct evidence for GPR41/GPR43‐mediated signaling in our model is lacking, and further studies are needed to establish causality. In addition to propionic acid, butyric acid, another major SCFA produced by gut microbial fermentation, has been reported to influence skeletal muscle homeostasis. Previous studies have shown that butyric acid can activate protein synthesis, inhibit oxidative stress, and suppress autophagy, with its levels positively correlated with muscle mass and function (Tang et al., 2022; Xie et al., 2026). In the present study, butyric acid levels were lower in males (MH group) than in females (FH group) in both serum and colonic contents. These findings suggest that reduced butyric acid levels may also contribute to the sexual dimorphism observed in obesity‐induced muscle atrophy under HFHC diet feeding. Notably, skeletal muscle transcriptomic analysis revealed that, among the mechanistic pathways associated with muscle atrophy, male mice primarily exhibit alterations in skeletal muscle function through energy metabolism, whereas female muscle atrophy is more closely associated with muscle protein metabolism and breakdown. This supports that, under HFHC dietary conditions, male mice are more susceptible than females to mitochondrial ETC dysfunction‐induced impairment of muscle energy metabolism, which further promotes obesity‐related muscle atrophy.

While the findings of this study are encouraging, several limitations must be acknowledged. First, the observed muscle atrophy is closely associated with sex hormones, and the potential confounding effects of estrogen and androgens could not be fully excluded. Future studies incorporating gonadectomy (e.g., ovariectomy or orchiectomy) would help clarify the hormonal influence. Second, the current study design does not permit causal inference regarding the relationship among gut microbiota, mitochondrial oxidative phosphorylation, and skeletal muscle atrophy. Future research employing fecal microbiota transplantation would be valuable to explore the potential causal link between gut microbiota and muscle atrophy via the gut‐muscle axis. Finally, the mechanistic investigation in this study remains primarily at the level of gene expression and has not yet extended to the protein and functional levels. Therefore, future research should perform in‐depth quantitative analyses at the protein level and adopt multi‐omics approaches to more comprehensively elucidate the potential mechanisms underlying sex dimorphism in obesity‐induced muscle atrophy.

In summary, this study confirms that female mice have a greater capacity to maintain homeostatic balance against HFHC diet‐induced muscle atrophy. This sex‐based difference is mainly attributed to variations in mitochondrial dysfunction, which arise from differing degrees of systemic metabolic dysregulation between males and females. Notably, male mice exhibited a more pronounced decrease in Cx‐III and Cx‐IV activities compared to females, leading to reduced energy production, respiratory inhibition, and exacerbated oxidative stress in mitochondria. In addition, male mice experienced more severe metabolic dysregulation, including gut microbiota dysbiosis that resulted in decreased SCFA production. In contrast, female mice maintained better intestinal physiological function during long‐term HFHC feeding by suppressing harmful bacterial proliferation and significantly enriching beneficial taxa with compensatory and reparative capacities. This study reveals that HFHC diet‐induced muscle atrophy exhibits sex‐dependent differences, and that mitochondrial energy metabolism is closely associated with skeletal muscle quality. These findings provide a theoretical basis for the targeted prevention of obesity‐related muscle atrophy.

## AUTHOR CONTRIBUTIONS

**Tingyi Mo:** Data curation; formal analysis; investigation; methodology; software. **Shan Wang:** Data curation; formal analysis; investigation. **Zhixin Li:** Investigation; methodology; validation. **Haobin Xu:** Conceptualization; data curation; formal analysis; investigation; methodology. **Xiaokang Liufu:** Methodology; software; validation. **Ya Wang:** Conceptualization; data curation; investigation; methodology; resources. **Nageena Qayyum:** Formal analysis; investigation; methodology. **Honghui Guo:** Conceptualization; data curation; funding acquisition; project administration; supervision.

## FUNDING INFORMATION

This study was funded by the National Natural Science Foundation of China (82273622).

## CONFLICT OF INTEREST STATEMENT

The authors declare no competing interests.

## Supporting information


**Table S1:** Composition of experimental diets.
**Table S2:** Primers used in the study.
**Table S3:** Effects of sex, diet, and their interaction on biochemical, skeletal muscle function, and histological outcomes in mice.
**Figure S1:** Analysis of DEGs in the GAS of four groups of mice. (a) Volcano plots displaying DEGs comparing group MH to group MC, group FH to group FC, and group FH to group MH. DEGs were identified using threshold parameters of fold change >1.2 or <0.83 and *p*‐value <0.05. (b) Profiles of upregulated and downregulated DEGs in the three comparison schemes. (c) Venn diagram showing the overlap among the three comparison schemes. (d) PCA analysis illustrating the separation of DEGs among the four groups. (e–g) GO enrichment analysis bar charts of the top 10 significantly enriched DEGs in MH versus MC, FH versus FC, and FH versus MH, including three categories: cellular components, biological processes and molecular functions. Each category displays the top 10 significantly enriched terms. (H–J) Bubble plots of the top 20 KEGG enrichment analyses of DEGs in MH versus MC, FH versus FC, and FH versus MH. Results are expressed as mean ± SD from samples of five mice per group. *p < 0.05, **p < 0.01.

## Data Availability

The analyzed data sets generated during the present study will be provided by the corresponding author on reasonable request.
