# Supplementary material for: Male mice are more susceptible than females to obesity‐induced muscle atrophy when fed a high‐fat, high‐cholesterol diet
Source: Physiol Rep. 2026 Oct 30;14(21):e71158. doi: 10.14814/phy2.71158 (PMC13722904; doi:10.14814/phy2.71158)
Supplement: Supplementary file 1 [file PHY2-14-e71158-s001.docx]

**Sup****plementary Tables**

**Table S1.** Composition of experimental diets.

| **Ingredients** | **Normal chow diet** | | **HFHC diet** | |
| --- | --- | --- | --- | --- |
|  | **Gram** | **Kcal** | **Gram** | **Kcal** |
| Casein | 200 | 800 | 209.8 | 839 |
| L-Cystine | 3 | 12 | 3 | 12 |
| Corn Starch | 381 | 1524 | 98 | 393 |
| Maltodextrin 10 | 100 | 400 | 117 | 466 |
| Sucrose | 120 | 480 | 201 | 805 |
| Cellulose | 75 | 0 | 58 | 0 |
| Soybean Oil | 50 | 450 | 29 | 262 |
| Lard | 0 | 0 | 207 | 1862 |
| Minerta Mix S10026 | 50 | 0 | 12 | 0 |
| DiCalcium Phosphate | 9 | 0 | 15 | 0 |
| Calcium Carbonate | 0 | 0 | 6 | 0 |
| Potassium Citrate, 1 H_2_O | 0 | 0 | 20 | 0 |
| Vitamin Mix V10001 | 10 | 40 | 12 | 47 |
| Choline Bitartrate | 2 | 0 | 2 | 0 |
| Cholesterol | 0 | 0 | 10 | 0 |
| Total | 1000 | 3706 | 1000 | 4686 |
| Kcal/Gram | 3.7 | | 4.7 | |

**Table S2.** Primers used in the study.

| **Gene Name** | **Forward Primer** | **Reverse Primer** | **Accession number** | **Amplicon size (bp)** |
| --- | --- | --- | --- | --- |
| *Fbox32* | 5′-CTTCTCGACTGCCATCCTGGAT-3′ | 5′-TCTTTTGGGCGATGCCACTCAG-3′ | NM_026346.3 | 107 |
| *Trim63* | 5′-TACCAAGCCTGTGGTCATCCTG-3′ | 5′-ACGGAAACGACCTCCAGACATG-3 | NM_00139048.2 | 133 |
| *Gapdh* | 5′-AGAAGGTGGTGAAGCAGGCATC-3′ | 5′-CGAAGGTGGAAGAGTGGGAGTTG-3′ | NM_001289726.2 | 111 |

**Table S3.** Effects of sex, diet, and their interaction on biochemical, skeletal muscle function, and histological outcomes in mice.

| Variable | *P* value | | |
| --- | --- | --- | --- |
|  | Sex | Diet | Sex × Diet |
| TG (mmol/L) | 0.792 | 0.055 | 0.529 |
| TC (mmol/L) | 0.258 | 0.001 | 0.345 |
| HDL-C (mmol/L) | 0.168 | < 0.001 | 0.153 |
| LDL-C (mmol/L) | 0.032 | < 0.001 | 0.001 |
| AST (U/L) | 1.000 | 0.001 | 0.078 |
| ALT (U/L) | 0.773 | < 0.001 | 0.873 |
| FBG (mmol/L) | 0.821 | 0.003 | 0.294 |
| FINS (mmol/L) | 0.019 | < 0.001 | 0.013 |
| HOMA-IR | 0.498 | < 0.001 | 0.093 |
| Forelimb grip strength (N/g) | 0.200 | < 0.001 | 0.093 |
| Four-limb grip strength (N/g) | 0.055 | < 0.001 | 0.074 |
| Rotarod test (s) | 0.042 | 0.003 | 0.093 |
| Y-maze distance traveled (m) | 0.728 | 0.001 | 0.183 |
| Y-maze arm entry (n) | 0.489 | < 0.001 | 0.263 |
| Open field distance traveled (m) | 0.750 | 0.039 | 0.161 |
| GAS index (%) | 0.988 | 0.008 | 0.002 |
| Muscle fiber cross-sectional area (μm^2^) | 0.095 | < 0.001 | 0.019 |
| F-actin fluorescence intensity (Fold change) | 0.429 | < 0.001 | 0.003 |
| *Fbxo32* | 0.451 | < 0.001 | 0.172 |
| *Trim63* | 0.096 | < 0.001 | 0.215 |
| Cx-I (U/mg prot) | 0.463 | < 0.001 | 0.488 |
| Cx-II (U/mg prot) | 0.219 | 0.694 | 0.694 |
| Cx-III (U/mg prot) | 0.049 | < 0.001 | 0.873 |
| Cx-IV (U/mg prot) | 0.018 | 0.002 | 0.001 |
| ROS (AU/mg prot) | 0.821 | 0.060 | 0.753 |
| T-SOD (U/mg prot) | 0.046 | < 0.001 | 0.017 |
| ATP (μmol/mg prot) | 0.030 | 0.001 | 0.345 |
| Acetic acid in serum (μg/mL) | 0.114 | 0.002 | 0.338 |
| Propionic acid in serum (μg/mL) | 0.598 | < 0.001 | 0.462 |
| Butyric acid in serum (μg/mL) | 0.012 | < 0.001 | 0.011 |
| Valeric acid in serum (μg/mL) | 0.005 | 0.036 | 0.048 |
| Total SCFAs in serum (μg/mL) | 0.048 | < 0.001 | 0.188 |
| Acetic acid in colonic contents (mg/g) | 0.132 | 0.018 | 0.172 |
| Propionic acid in colonic contents (mg/g) | 0.851 | < 0.001 | 0.036 |
| Butyric acid in colonic contents (mg/g) | 0.243 | 0.007 | 0.074 |
| Valeric acid in colonic contents (mg/g) | 0.794 | < 0.001 | 0.959 |
| Total SCFAs in colonic contents (mg/g) | 0.243 | 0.005 | 0.074 |

**Supplementary Figures**

**Fig. S1 Analysis of DEGs in the GAS of four groups of mice.** (A) Volcano plots displaying DEGs comparing group MH to group MC, group FH to group FC, and group FH to group MH. DEGs were identified using threshold parameters of fold change > 1.2 or < 0.83 and *P*‐value < 0.05. (B) Profiles of upregulated and downregulated DEGs in the three comparison schemes. (C) Venn diagram showing the overlap among the three comparison schemes. (D) PCA analysis illustrating the separation of DEGs among the four groups. (E-G) GO enrichment analysis bar charts of the top 10 significantly enriched DEGs in MH *vs.* MC, FH *vs.* FC, and FH *vs.* MH, including three categories: cellular components, biological processes and molecular functions. Each category displays the top 10 significantly enriched terms. (H-J) Bubble plots of the top 20 KEGG enrichment analyses of DEGs in MH *vs.* MC, FH *vs.* FC, and FH *vs.* MH. Results are expressed as mean ± SD from samples of five mice per group. **P* < 0.05, ***P* < 0.01.
